# Temperature‐regulated gold nanoparticle sensors for immune chromatographic rapid test kits with reproducible sensitivity: a study

**DOI:** 10.1049/nbt2.12024

**Published:** 2021-03-03

**Authors:** Prince Manta, Suresh Chandra Singh, Aman Deep, Deepak N. Kapoor

**Affiliations:** ^1^ School of Pharmaceutical Sciences Shoolini University Bajhol Solan Himachal Pradesh India; ^2^ Seloi Healthcare Pvt. Ltd Goregaon West Mumbai India

## Abstract

Immune‐chromatographic kits are being used since several years in the rapid detection of infectious diseases. It is also called the lateral flow technique, and is used for antigen or antibody detection. There are a series of steps involved in the development of these immune‐chromatographic test kits. Still, the preparation of gold nanoparticles (AuNPs) is an important quality variable for the immune‐chromatographic test kit sensitivity. The immune chromatographic test must be specific in detection for specific antigen and antibody; this implies that the test kit should not show a false result. Secondly, the test kit should be sensitive enough to give a readable result, and the intensity of the test line should increase or decrease with the concentration of an analytic sample. Various factors can influence the performance of a test. Temperature differences in AuNPs preparation can alter the assay kinetics and contribute to assay variability. Other factors such as assay components, manufacturing processes and reagent variation also contribute to assay precision and accuracy. It is important to note that assay reproducibility is the combined effect of individual sources of variability. The authors have synthesized AuNPs by immediately controlling the reaction temperature. Different batches of Malaria rapid test kit were developed and the test kit sensitivity was analysed. It was found that test kits designed with temperature‐controlled AuNPs sensor had reproducible uniformity in terms of batch to batch sensitivity than AuNPs synthesized by conventional Turkevich and Fern process.

## 1 INTRODUCTION

The healthcare system of a country is largely dependent on the precise and accurate diagnosis of the disease. Precise and accurate diagnosis not only saves the life of the patients but also reduces the overall medical burden of the country by decreasing the time of bed occupancy in critical care and the time the doctors spent on the patients [1]. In a developing country, unlike a developed country where most of the test is carried out in the central facility by specialized and highly trained people, there is an immediate need for test which requires minimal infrastructure, skilled human resources and low budget [2].

The limitation mentioned above makes point‐of‐care test (POCT) extremely useful as it allows reduction in time and efforts involved in sample transportation and processing of the sample. This, in turn, reduces the time between diagnosis and appropriate medical intervention.

One of the most popular techniques for POCT is lateral flow assay (LFA) or more exactly lateral flow immune assay [3, 4, 5]. Features which make lateral flow assay as a widely used and accepted diagnostic platform are ease of use, low cost of manufacturing, robustness, and no or minimal need of skill to perform and interpret the results. These features are responsible for making this technique as a platform technology for various over the counter diagnostic products like pregnancy test kit [6, 7]. These immunoassays employ the inherent sensitivity, specificity and binding affinities of antibodies towards respective antigens to detect their presence in a sample. Samples typically tested include urine, whole blood, plasma, serum, saliva and respiratory fluids. Immunoassay signals typically come from the accumulation of a labelled antibody at the binding site for the antigen on a substrate. Typical antibody labels may be fluorescent molecules, nano‐ or micro‐particles, or enzymes.

The working/principle of lateral flow immunoassay is based on sandwich assay [8]. Antibodies in the conjugate pad tend to attach to the antigens present in the sample solution and form a complex, which gets attached to test line antibodies. The excess of labelled antibodies binds to the antibodies present in the control line. There are many factors involved in causing variation in the quality of LFA components like sample pad, nitrocellulose membrane, conjugate pad and gold nanoparticles. A gold nanoparticle plays an important role in LFA as it is used in conjugation. There are many research articles and papers that define the work of gold nanoparticles on its physical and chemical interactions. Some of these are ionic interaction between gold nanoparticles and antibodies (as both are oppositely charged), hydrophobic interaction, and conducting principle between the electrons of gold and an amino acid sulphur group of the antibody [9]. Those mentioned above were the three major interactions but there are multiple other ways of interaction through thiol derivatives, bifunctional linkers and usage of adapter molecules like streptavidin and biotin [10, 11].

Gold nanoparticle ranging from 1 to 100 nm in size has been synthesized as reported by Turkevich et al. [12, 13]. Similarly, Fern et al. reported a refined process of preparation of gold nanoparticles with particle size control, simplicity, scalability and improved stability [14]. Despite of all the advantages, one of the major problems faced in lateral flow method is the lack of reproducibility. This not only increases manufacturing cost but also exposes the patients at an enormous risk of incorrect diagnosis, especially for severe diseases such as HIV, Hepatitis B, HCV and COVID‐19.

It was observed by Grigoris et al. that size of gold particles can be controlled by the temperature during particle synthesis [15]. Higher the temperature, higher is the rate of nanoparticle formation and uniformity in the size. They bifurcated the synthesis into three stages:

- During the time of incubation, a small increase in the hydrodynamic radius of the particles is observed when compared to the radius of the net polymer, which is about 10 nm.
- In the subsequent step, nanocluster property should be intensified by increasing the formation of Au metal nanoparticles [16]. In particular, the intensity increased by a factor of several hundred during this nanoparticle development stage.
- Polydispersity index (PDI) and Hydrodynamic radius factors to be slightly increased so that there can be an increase in the intensity of scattered light, at this point the particles tend to remain stable and no variation is observed in the factors like PDI, light scattering intensity and hydrodynamic radius.

Now comparing with the conventional method, the gold nanoparticle after boiling in citrate solution turns to wine‐red at room temperature which comes in the b and c stages as mentioned above, which characterize the size and uniformity of the particles. So in the proposed modified method, there will be incorporation of b and c stages immediately after the boiling, and the effect is looked in all the immune chromatographic test kits batches.

## 2 MATERIALS

Gold(III) chloride was purchased from Sigma Aldrich, monosodium hydrogen phosphate, disodium hydrogen phosphate, sodium chloride, BSA (bovine serum albumin), trisodium citrate and distilled water were purchased from Merck. Malaria *P. vivax* LDH Monoclonal antibody and Goat anti‐mouse IgG were purchased from Arista Biologicals, Inc. While PVC backing from were purchased from Future Care Diagnostic and nitrocellulose membranes, sample pad, conjugate pad, absorbent pad were purchsed from MDI, Ambala. Bio Dot XZ1010 (Biotron) was used for coating antibody on membrane. The Delsa™ Nano Submicron Particle Size Analyzer of Beckman Coulter, California was used for analysing AuNPs diameters. Ultraviolet‐visible spectrophotometer 1900i of Shimadzu, Japan was used to measure optical density and absorbance maxima.

## 3 METHOD

### 3.1 Preparation of gold nanoparticles

Colloidal gold particles were prepared using citrate reduction method (Frens G 1973). An aqueous solution of chloroauric acid 0.01 (v/v) % was prepared in distilled water with constant stirring.

Optical density of the solution was set between 0.7 and 470 nm wavelength. This solution was refluxed at a temperature of 95°C in 760.2 mmHg for 30 min to boiling point in temperature controlled chamber and further reduced by trisodium citrate with constant stirring. Colour of the solution starts changing from yellow to black and then to pink [17]. In the classical Frens protocol, the solution is kept at room temperature to attain the third phase. The authors follow the modified process in which, once a pink colour was developed, then the reaction was immediately stopped by immediately taking off the solution from heat and cooling in an ice bath. However, there are multiple factors which influence the diameters of the nanoparticles in classical Turkevich and Fern protocols. The various factors affecting nanoparticles are the molar ratio of chloroauric acid, proportional reaction batch size, and the concentration of trisodium citrate and effect of reaction temperature [18, 19]. Apart for temperature, others factors are easily validated before reaction starts, but the temperature needs to be controlled during reaction, that changes the latent heat during water boiling and can change the size of the nanoparticles to 3 nm approximately. At the same time if the reaction temperature goes down, smaller sized nanoparticles were produced. When the reaction temperature decreases less than 90°C, it causes a decrease in reduction rate and overall the nucleation rate. The latent heat in reaction solvents is one way to control nucleation and growth in the synthesis of gold nanoparticles [18, 19]. In our modified process, immediate reaction ceasing was done as a pink colour develops by cooling down the reaction temperature on ice bath. This method cannot alter the latent heat during water boiling leading to the development of nanoparticles with less size deviation.

Gold nanoparticles thus obtained from both the process, can be stored for several months at 2–8°C for further protein conjugation. Gold nanoparticles characterization was done using UV‐Visible spectrometer by scanning in the visible range and diameters analysis by zeta seizers.

### 3.2 Conjugation of colloidal gold with pLDH monoclonal antibodies

For conjugation pH of colloidal gold, the solution was adjusted to pH 7.5 with 0.1 M potassium carbonate buffer. The pLDH monoclonal antibody was added to get a final concentration of 250 µg /ml followed by incubation at 4°C for 30 min. Conjugate was then centrifuged at with relative centrifugal force (RCF) of 7000 × *g* for 30 min. The supernatant was discarded, and the pellet obtained was washed twice with 100 ml of a solution followed by centrifuge at 4°C with RCF of 7000 × *g* for 30 min each time. Finally, the pellet was diluted to concentrate it 10 times in 10 mM PBS buffer of pH 7.5. Conjugate can be stored up to a year at 4°C.

### 3.3 Preparation of immunochromatographic strip

Paek et al.'s (Methods 22) method was used for the construction of immunochromatographic strips [20]. Gold colloidal‐antibody was diluted to 50 µg/ml and was coated on non‐woven cloth sheets by dipping/spraying. Glass fibre for sample pad was treated with (1 ml/cm^2^) 10 mM PBS pH 7.0, 0.5% ovalbumin, 0.01% tween‐20, 0.2‰ NaN_3_ followed by drying at 37°C and cutting into 30 × 2 cm strips. The nitrocellulose membrane was assembled on a backing laminate. pLDH antibody in PBS buffer (150 µg/ml) was coated on a nitrocellulose membrane using Biodot XZ1010 for test line (Pv) along with goat anti‐mouse IgG antibody in PBS buffer (0.4 mg/ml) as the control (C) line. These laminates were then dried at 25°C for 30 min. Treated sample pad, conjugate pad and absorbent pad were then laminated on backing and cut into individual strips of 3 mm using a guillotine cutter. Strips were then assembled into plastic housing.

### 3.4 The procedure of blood samples detection

The test kits developed from normal and modified methods were tested using same blood specimen of *P. vivax* (Pv) of concentration 40 parasites/μl to compare the band intensity in repeated batches. The test kits developed from modified menthods were additionally tested using *P. Falciparum* (Pf) of concentration 40 parasites/μl for specificity. About 5 µl of blood malaria Pv & Pf antigen specimens was added to sample well followed by addition of three drops of a buffer. After 5 min, colour signals were developed and results were judged by the development of lines using RGB and HSV colour models. Pv line developed along with control test was considered positive for *P. vivex, P. malariae/P. ovale*. If no line developed, then the test was considered invalid.

## 4 RESULTS

### 4.1 UV analysis of gold nanoparticles

**FIGURE 1:**
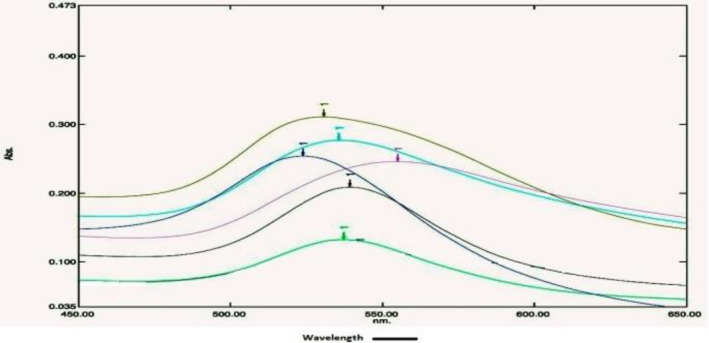
Visible spectra and λ_max_ overlay of six different gold nanoparticles synthesized from of conventional method. The spectra were obtained by scanning nanoparticles between wavelength 650 and 450 nm. A spectrum shows wide variations in absorbance at particular wavelength (λ_max_)

**FIGURE 2:**
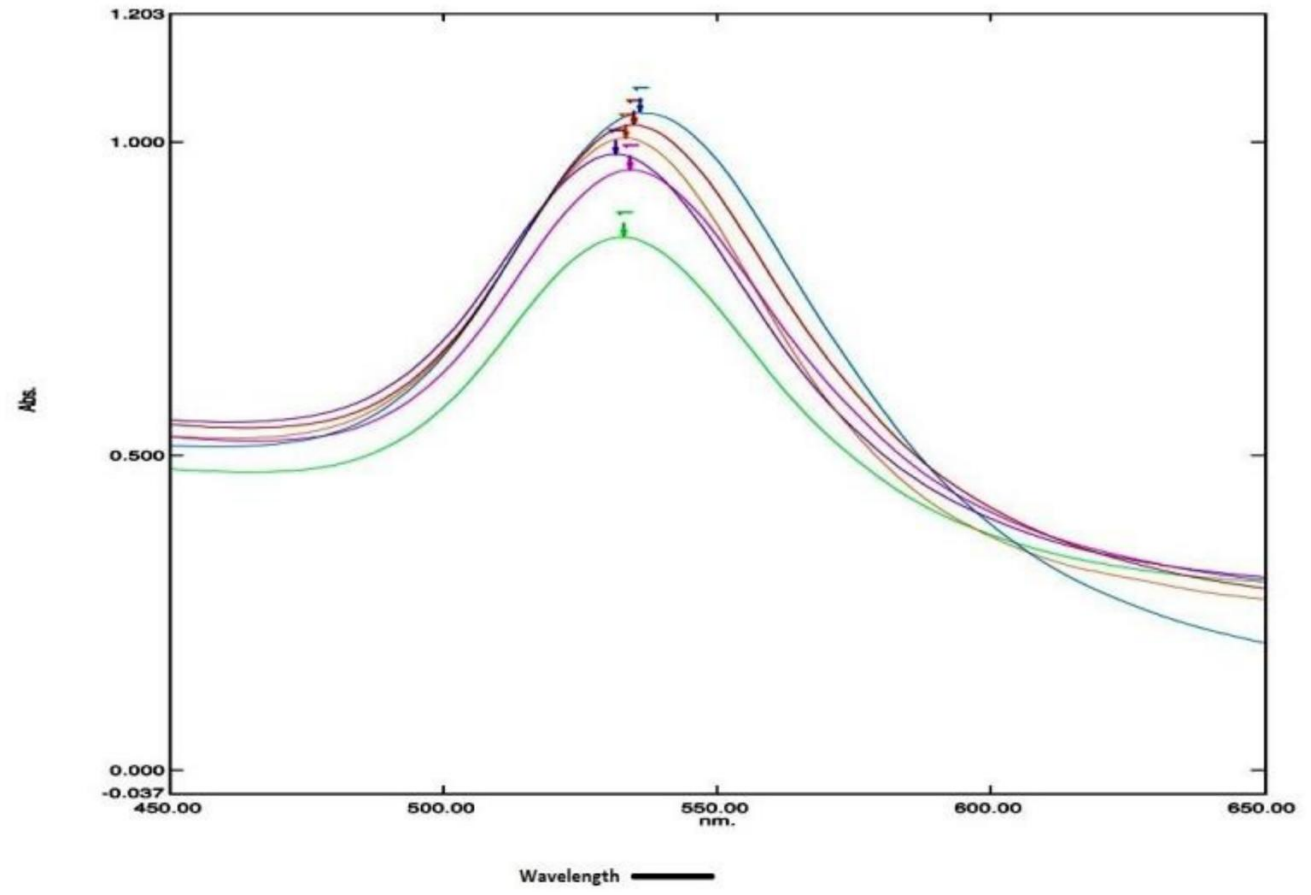
Visible spectra and λ_max_ overlay of six different gold nanoparticles synthesized from the modified method. The spectra were obtained by scanning nanoparticles between wavelength 650 and 450 nm. A spectrum shows less variation in absorbance at a particular wavelength (λ_max_)

The spectrum measurement of gold nanoparticles synthesized by the conventional and modified process was done in a visible range of wavelength 650–450 nm taking water as blank. A UV‐Vis spectrometer is used to determine the optical density (absorbance) at a particular wavelength (λ_max_). The spectrophotometric scan of the gold nanoparticle in the normal fern process is very inconsistent having λ_max_ in between 524.7 and 557.0 nm and absorbance between 0.108 and 0.325. The conventional process showed lots of variation both in spectra and in the λ_max_ (Figure 1 and Table 1). However, the modified method showed almost the same spectra and λ_max_ in every batch (Figure 2 and Table 2). The spectrophotometric scan of gold nanoparticle of modified method is very consistent and having λ_max_ in between 531.4 and 535.8 nm and absorbance is between 0.840 and 1.046.

**TABLE 1:** Spectrum scans observation of gold nanoparticles synthesized by the conventional method

Reaction replication number	Gold nanoparticles spectra	Wavelength (λ_max_)	Optical density (Abs)
1	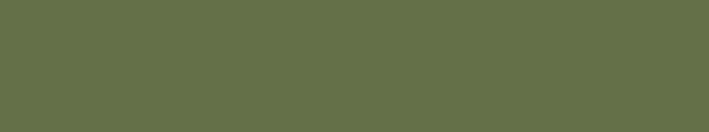 AuNPs RT ‐ 1	537.2	0.325
2	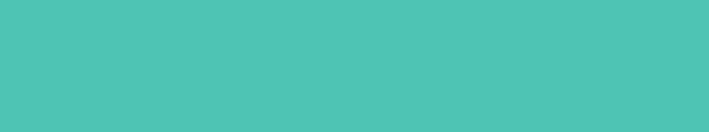 AuNPs RT ‐ 2	542.5	0.274
3	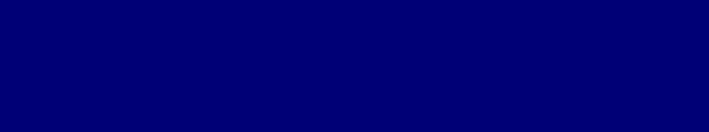 AuNPs RT ‐ 3	524.7	0.239
4	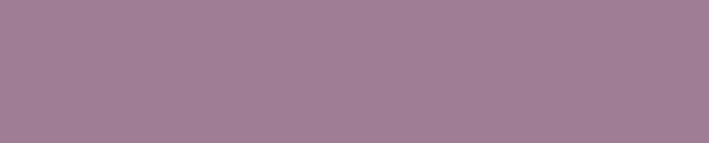 AuNPs RT ‐ 4	557.0	0.221
5	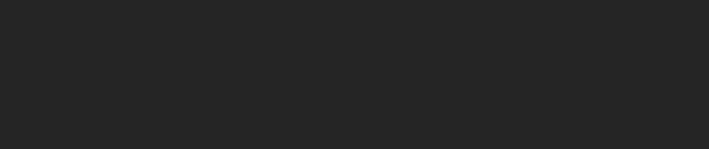 AuNPs RT ‐ 5	544.6	0.184
6	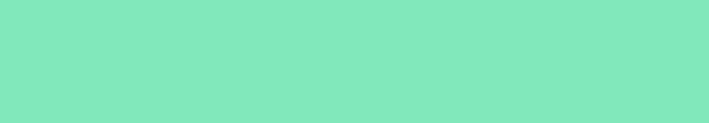 AuNPs RT ‐ 6	541.8	0.108

Abbreviation: AuNP, gold nanoparticle.

**TABLE 2:** Spectrum scan observation of gold nanoparticles synthesized by modified method

Reaction replication number	Gold nanoparticles spectra	Wavelength (λ_max_) (nm)	Optical density (Abs)
1	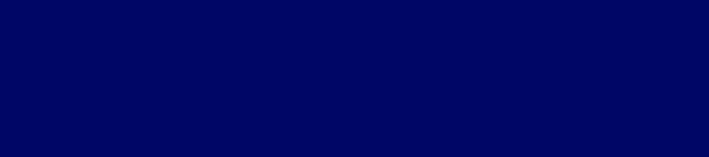 AuNPs RT – 1	535.8	1.046
2	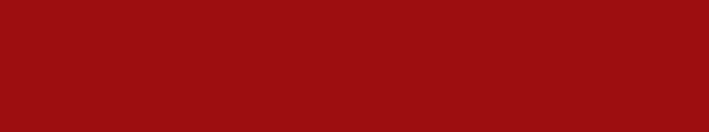 AuNPs RT – 2	534.0	1.026
3	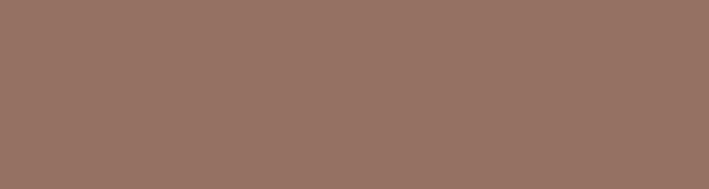 AuNPs RT – 3	533.4	1.006
4	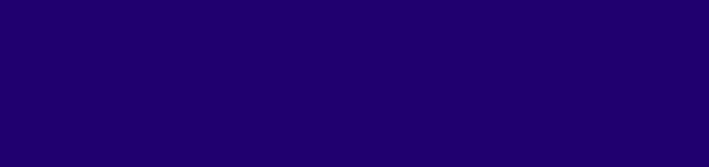 AuNPs RT – 4	531.4	0.980
5	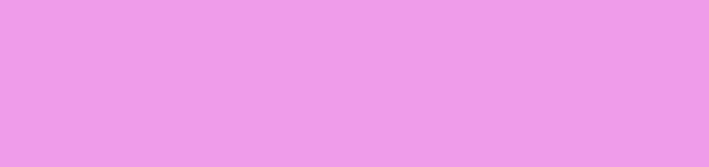 AuNPs RT – 5	534.2	0.955
6	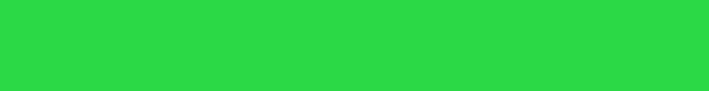 AuNPs RT – 6	532.8	0.840

Abbreviation: AuNP, gold nanoparticle.

As explained, the colloidal gold nanoparticles were prepared by reducing the chloroauric acid using trisodium citrate. In the modified process, reduction reaction is stopped once the pink colour was developed by keeping it in an ice bath from hot temperature. It helps in attaining the gold nanoparticle in a particular size of 20–40 nm.

### 4.2 Particle size analysis of gold nanoparticles

**FIGURE 3:**
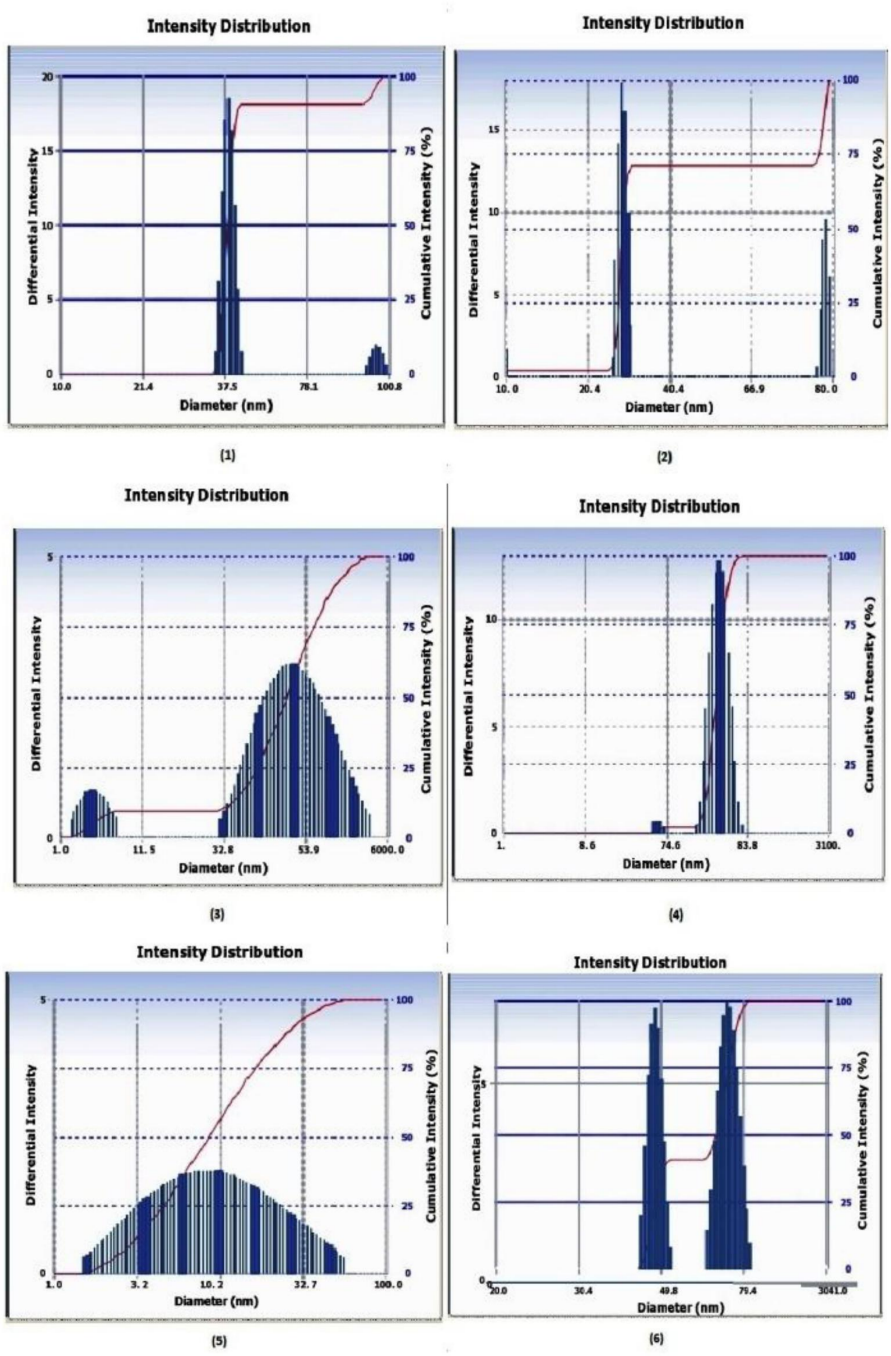
The figure represents the graph of the gold nanoparticles synthesized form the conventional process. The graph numbers 1–6 represent the gold nanoparticle's diameters sizes synthesized in six different batches

The sizes of six different batches of gold nanoparticles prepared by conventional and modified method were analysed by zeta sizer. In the conventional method, if reaction was not stopped immediately by cooling, two different particle size distributions were observed in a single reaction (batch) (Table 3 and Figure 3). Also the nanoparticles thus produced have wide range of variations in diameters during reaction replication. In this case, the protein conjugation with nanoparticles leads to uneven surface binding causing less uniformity in colour intensity of test and control bands. Additionally, batch to batch variation of the kits is also very high.

**TABLE 3:** Average particle size of gold nanoparticles prepared by conventional and modified process

Reaction replication number	Average particle size of nanoparticles prepared by conventional method (nm)	Average particle size of nanoparticles prepared by modified method (nm)
1	Size A‐ 37.5	32.2
	Size B‐ 98.3	
2	Size A‐ 25.5	24.8
	Size B‐ 78.4	
3	Size A‐ 2.8	37.3
	Size B‐ 50.4	
4	Size A‐ 59.4	39.1
	Size B‐ 81.8	
5	12.8 nm	25.6
6	Size A‐ 48.1	30.6
	Size B‐ 74.6	

**FIGURE 4:**
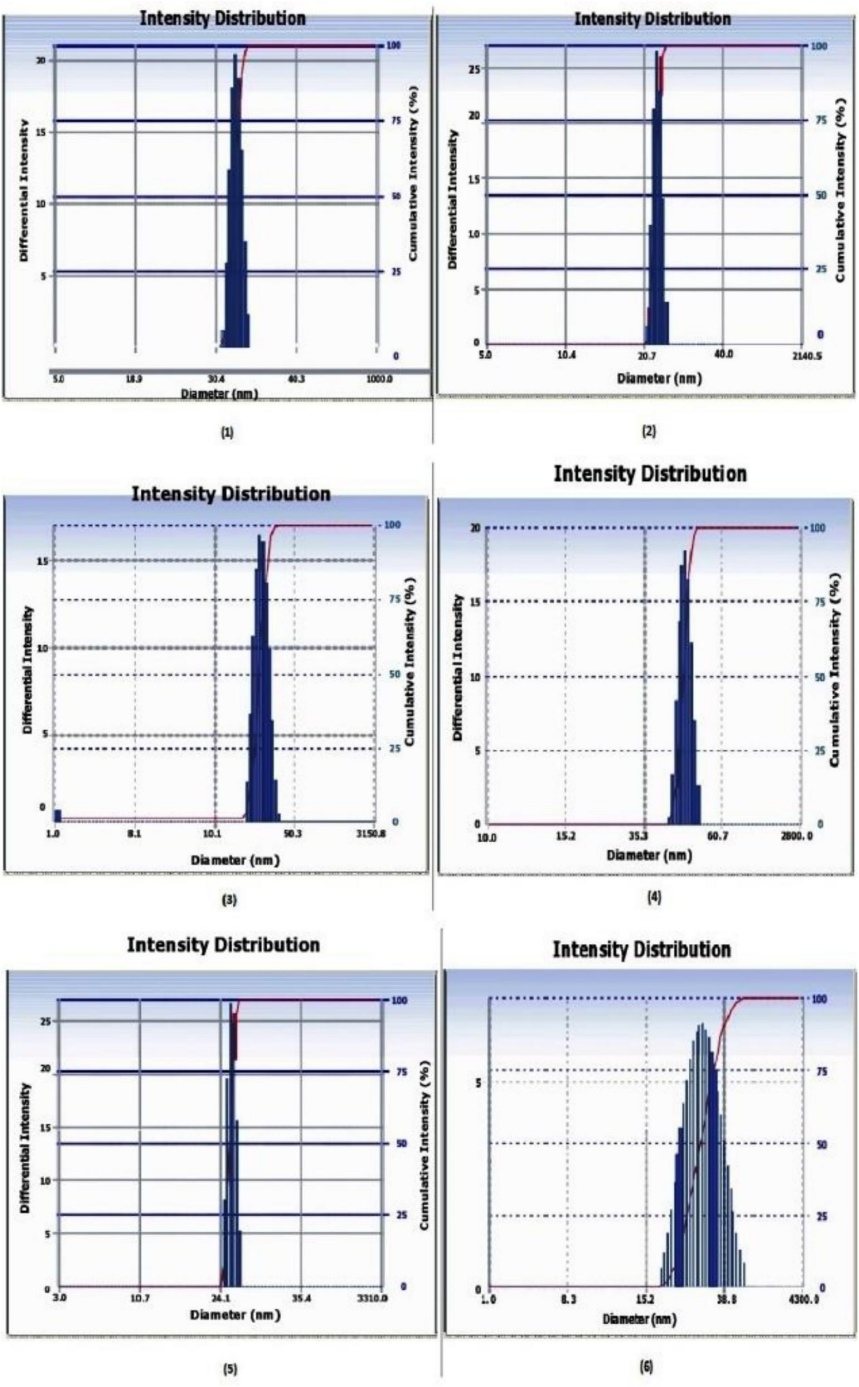
The figure represents the graph of gold nanoparticles synthesized form the modified process. The graph number 1–6 represent the gold nanoparticle's diameter sizes synthesized in six different batches

The gold nanoparticles synthesized by a modified method produced nanoparticles with single and uniform particle size distribution ranging from 24.8 to 39.1 nm as indicated in Table 3 and Figure 4. In the modified process, nanoparticles had less variation in the particle diameter as compared with the conventional method and conjugation was uniform with less batch to batch variation in band intensity.

### 4.3 Comparison of kits prepared by conventional and modified process

**FIGURE 5:**
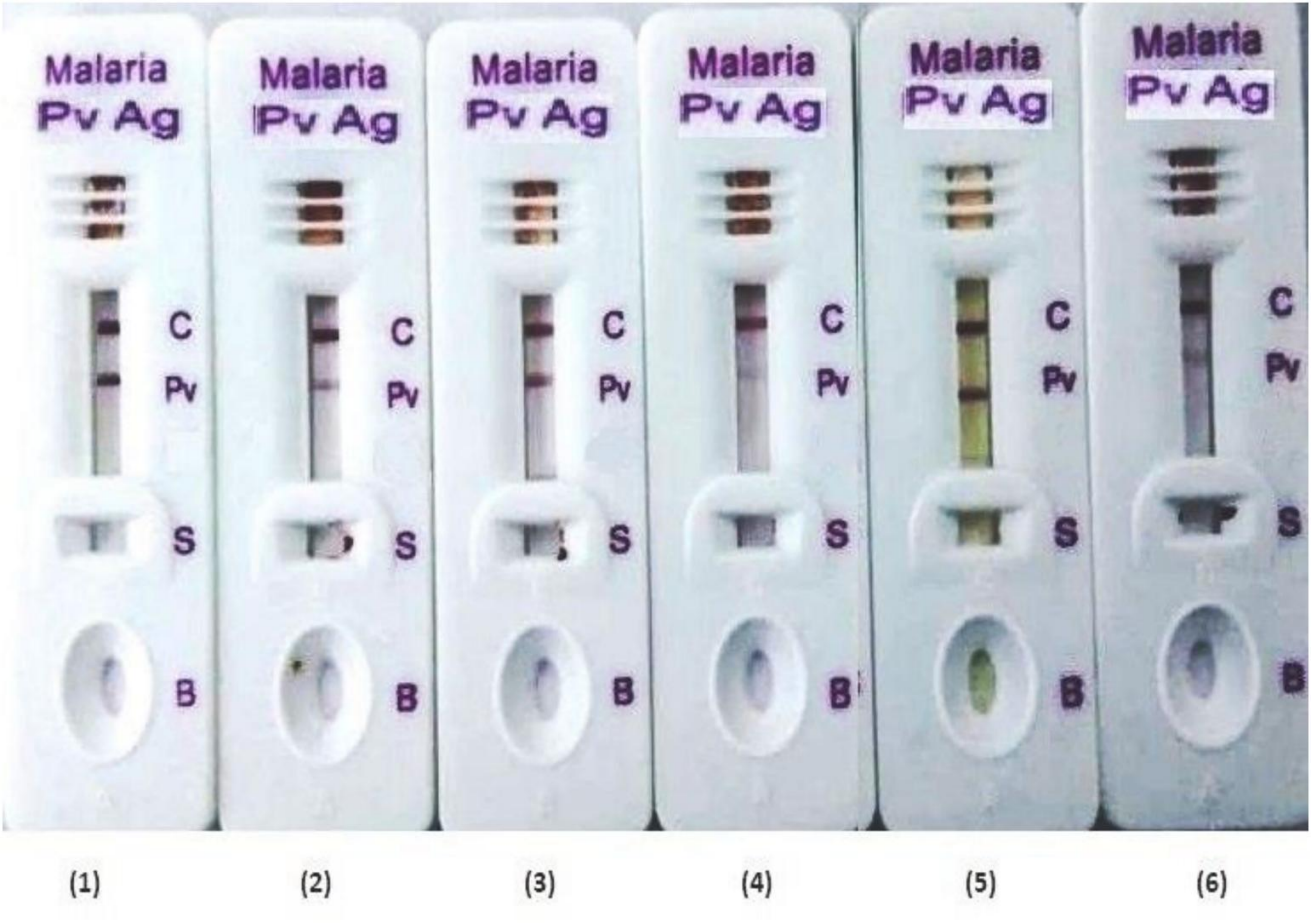
The figure represents the Malaria Pv Antigen kits test band intensity variation of six (1–6) different batches manufactured by conventional method. All the kits were tested using blood specimen of *P. vivex* (40 parasites/μl)

The Malaria Pv antigen positive samples were tested in six different batches developed from gold nanoparticles synthesized by conventional and modified process. The sample dispensed in the test was 5 μl and 3 drops of buffer (approx. 120 μl) in the test. The test kit developed by both conventional and modified methods showed limit of detection for *P. vivex* >4 parasites/μl of blood. The conventional method showed uniformity variation in the test band intensity in simultaneously taken batches. The test kits developed from conventional methods showed variations in terms of false negative results, weak positive to strong positive results when checked with same *P. vivex* of concentration 40 parasites/μl in simultaneously taken batches (Figure 5).

**FIGURE 6:**
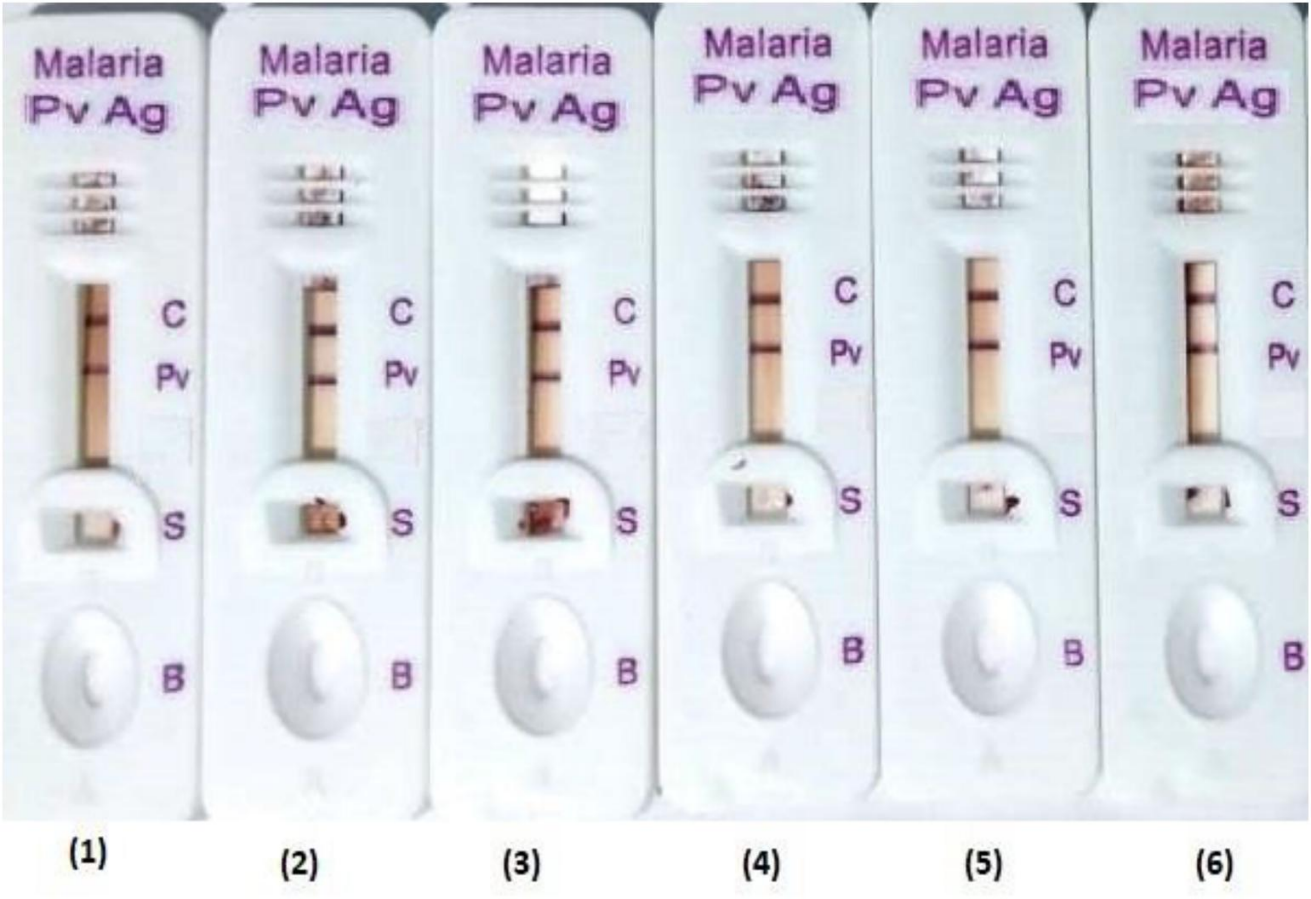
The figure represents the Malaria Pv Antigen kits test band intensity reproducibility of six (1–6) different batches manufactured by modified method. All the kits were tested using blood specimen of *P. vivex* (40 parasites/μl)

The test kits developed from modified methods showed equal strong positive test band intensity in simultaneously taken batches when compared using RGB and HSV colour models (Figure 6).

**FIGURE 7:**
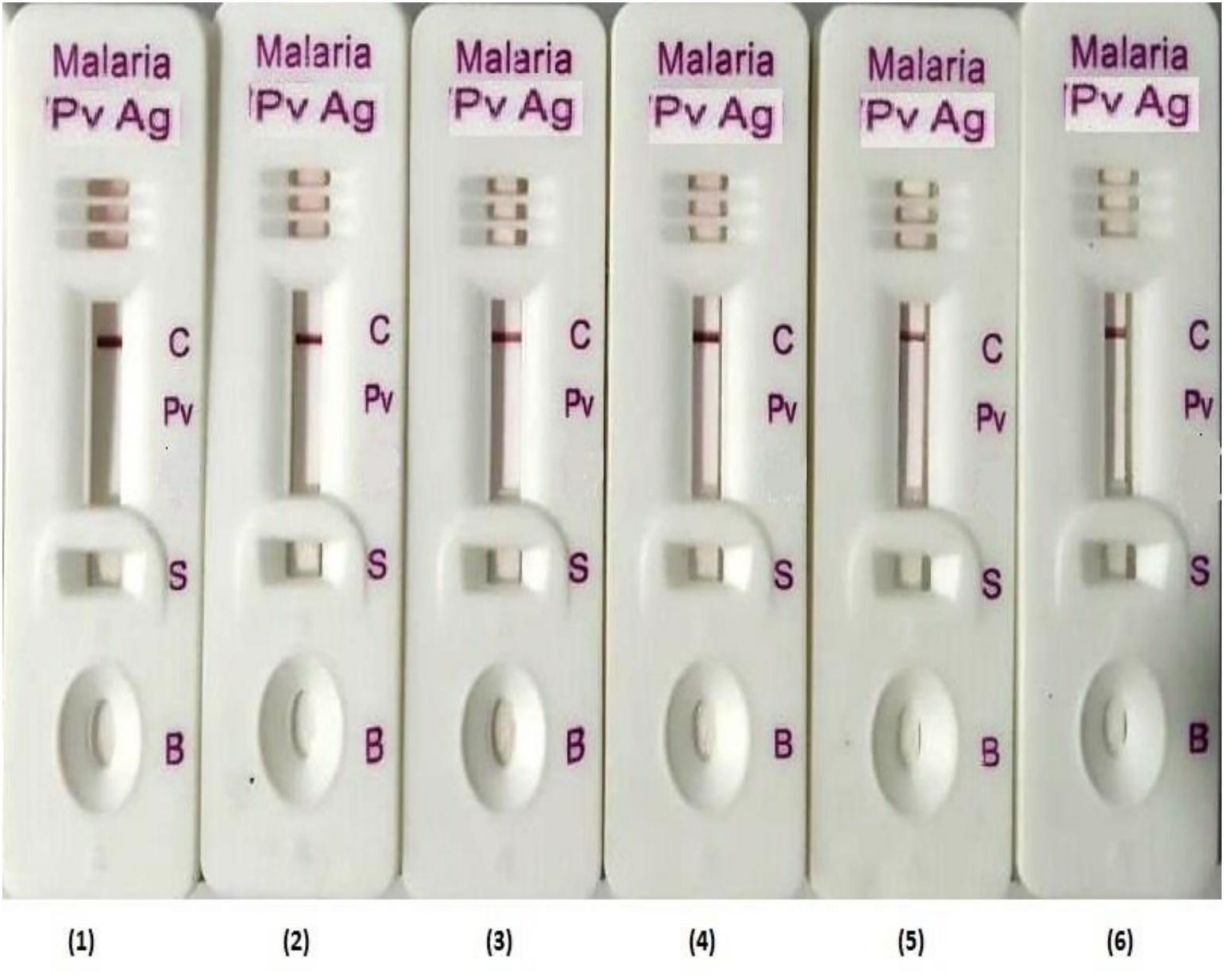
The figure represents the Malaria Pv Antigen kits control test results of six (1–6) different batches manufactured by modified method. All the kits were tested using blood specimen of *P. Falciparum* (40 parasites/μl)

The visual band of batches manufactured from modified process almost appeared on the same time (2 min) which was not the case with batches made from conventional process. The time of appearance of the visual band was much later than the modified process and was uneven. The test kits developed from modified process was additionally tested for its specificity with 5 μl of blood Malaria Pf *(Falciparum*) antigen specimens (40 parasites/μl) to find out test kits susceptibility for *P. Falciparum*. The test kits prepared using modified method was found to be efficient in terms of specificity (Figure 7)

## 5 CONCLUSION

Since many decades, the Turkevich and Ferns method is being used for the synthesis of AuNPs sensors without any significant modification for rapid immunochromatographic test kits. The method is also well‐suited and well‐adopted for its easy scale‐up and undoubtedly has consistent results. These methods were well accepted until the method is used for qualitative purpose and the regulatory scrutiny by the authorities was not very narrow. With new advances in the quantitative side of LFA, there is a consistent demand for the reproducible and very robust manufacturing process. The authors successfully show that there is a marked improvement in the gold nanoparticle synthesis with the modified method in terms of size distribution and results reproducibility. Gold nanoparticles prepared by modified method were found to be far more sensitive with uniform colour intensity and almost negligible background effect.
